# Single-cell RNA-seq analysis reveals that immune cells induce human nucleus pulposus ossification and degeneration

**DOI:** 10.3389/fimmu.2023.1224627

**Published:** 2023-08-10

**Authors:** Song Guo, Meijun Yan, Xinhua Li, Shuya Zhang, Zhong Liu, Kewei Li, Pengcheng Liu, Yanbin Liu, Guixin Sun, Qiang Fu

**Affiliations:** ^1^ Department of Spine Surgery, Shanghai Jiaotong University First People’s Hospital, Shanghai, China; ^2^ Department of Neurologic Surgery, Mayo Clinic, Rochester, MN, United States; ^3^ Department of Traumatology, Shanghai East Hospital, Tongji University School of Medicine, Shanghai, China

**Keywords:** single-cell RNA-seq analysis, IDD, nucleus pulposus, ossification, TNF-α

## Abstract

**Background and aims:**

Determining the transcriptomes and molecular mechanism underlying human degenerative nucleus pulposus (NP) is of critical importance for treating intervertebral disc degeneration (IDD). Here, we aimed to elucidate the detailed molecular mechanism of NP ossification and IDD using single-cell RNA sequencing.

**Methods:**

Single-cell RNA-seq and bioinformatic analysis were performed to identify NP cell populations with gene signatures, biological processes and pathways, and subpopulation analysis, RNA velocity analysis, and cell-to-cell communication analysis were performed in four IDD patients. We also verified the effects of immune cells on NP ossification using cultured NP cells and a well-established rat IDD model.

**Results:**

We identified five cell populations with gene expression profiles in degenerative NP at single-cell resolution. GO database analysis showed that degenerative NP-associated genes were mainly enriched in extracellular matrix organization, immune response, and ossification. Gene set enrichment analysis showed that rheumatoid arthritis signaling, antigen processing and presentation signaling were activated in the blood cell cluster. We revealed that stromal cells, which are progenitor cells, differentiated toward an ossification phenotype and delineated interactions between immune cells (macrophages and T cells) and stromal cells. Immune factors such as TNF-α, CD74 and CCL-3 promoted the differentiation of stromal cells toward an ossification phenotype *in vitro*. Blocking TNF-α with a specific inhibitor successfully reversed NP ossification and modified NP morphology *in vivo*.

**Conclusion:**

Our study revealed an increase in macrophages and T cells in degenerative NP, which induced stromal cell differentiation toward an ossification phenotype, and contributed to the identification of a novel therapeutic target to delay IDD.

## Introduction

Lumbar intervertebral disc degeneration disease (IDD) has become the main cause of lower back and leg pain (1). Lumbar fusion surgery is an effective treatment for patients with IDD who are refractory to conservative treatment. However, current lumbar fusion surgery has many complications, such as high nerve injury risk, increased radiation exposure, and accelerated degeneration of adjacent segments (2). In recent years, exogenous stem cell transplantation, which is the main method of cell therapy, has been shown to delay IDD, but there are some shortcomings, including short therapeutic efficacy, heterotopic ossification, and the risk of tumorigenesis (3). Therefore, the identification and activation of endogenous progenitor cells in degenerative intervertebral discs is another key strategy to enhance cell repair. Recently, a scRNA-seq study of five healthy intervertebral discs identified nine types of cells in healthy intervertebral discs and confirmed PDGFRA- and PROCR-positive cells as progenitor cells (4). However, cell heterogeneity, cell dynamics and interactions in the degenerative nuclear pulposus (NP) remain unclear.

Healthy intervertebral discs, which are the largest avascular tissues, have the anatomical privilege of immune silencing (5). Damage and rupture of the annulus fibrosus results in vascular growth and the infiltration of proinflammatory macrophages into the intervertebral disc to accelerate IDD (6). Coculture of macrophages (MCs) and bovine intervertebral discs significantly reduced the expression of proteoglycan and type II collagen in intervertebral discs and caused IDD (7). Additionally, plasmacytoid DCs were significantly increased in degenerative NP, presented antigens and initiated the immune response (8). Furthermore, Treg cells were significantly increased in degenerative NP, induced IDD and caused lower back pain by secreting IL-17 (9). These factors were individually studied, and there is still a lack of high-throughput sequencing data to investigate the effect of immune cells on NP degeneration. This study examined the effect of blood-derived immune cells on NP degeneration by using scRNA-seq analysis. Our preliminary analysis showed that the biological processes associated with the immune response and ossification were significantly enriched in degenerative NP tissue. NP ossification is often found in IDD patients and is a degenerative change in intervertebral discs. Although several studies have shown that NP ossification may be related to metabolic changes, inflammation triggered by trauma, and reduced nutrient supply (10), the detailed mechanism remains unclear. Therefore, we explored the effects and detailed molecular mechanism of immune cells on NP ossification in this study. This study provided an unbiased atlas of degenerative NP cells and shed light on diagnostic and therapeutic targets for NP ossification and IDD.

## Materials and methods

### Methods

#### Patients and samples

NP samples were obtained from patients with a diagnosis of IDD during surgery at Shanghai Jiaotong University First People’s Hospital. Research protocols involving human subjects were approved by the Ethics Committee of Shanghai Jiaotong University First People’s Hospital (2021SQ091). All participants provided written informed consent in accordance with the Declaration of Helsinki. All patients and the next of kin were fully legally competent and consented to the use of lumbar NP tissues and blood samples for research. The privacy rights of the patients or next of kin were always protected. The patients or the next of kin provided written informed consent to publish case details in this manuscript.

### Cell suspension preparation

NP samples from four patients in lumbar discectomy surgery were collected and minced with a razor blade into 1 mm^3^ fragments on a dish under aseptic conditions. NP fragments were suspended in 10 ml of 1×TrypLE Express and incubated in 37°C water bath with shaking for 30 min. Centrifuge the tissue at 500g for 5 minutes at room temperature and wash twice by adding 25 ml PBS. Discard the supernatant and add 10 mL of digestion buffer consisting of 2 mg/mL Collagenase type II (Sigma, c6885), 0.1mg/ml DNase I (Roche, 4716728001) in RPMI medium (Coring, 10-040-CV), and incubated in 37°C water bath with shaking for 4-6 hours. Terminate the enzymatic digestion when the tissue pieces have entirely disintegrated.

The suspension was passed through a 100 μm filter (Falcon, 3523260) and centrifuged (400g, 10 min, 4°C). Pelleted cells were resuspended in red blood cell lysis buffer (Solarbio, R1010) for 5 min at room temperature, followed by an additional filter step through a 40 μm filter (Falcon,3523240). Cells were collected by centrifugation (400g, 10 min, 4°C) and resuspended in PBS (BI, 02-024-1ACS) containing 0.04% BSA (Sigma, B2064).

Cells were manually counted by Trypan blue (Thermo, T10282) and AO-PI (LUNA, D23001) after each centrifugation (400g, 10 min, 4°C) and resuspended. Single cells were processed using Chromium Controller (10× Genomics) according to the manufacturer’s protocol.

### Single-cell RNA sequencing

By using Chromium Next GEM Single Cell 3′ Kit v3.1 (10x Genomics, 1000268) and Chromium Next GEM Chip G Single Cell Kit (10xGenomics, 1000120), we performed single cell 3′ gene expression profiling. The cell suspension was loaded onto the Chromium single cell controller (10x Genomics) to generate single-cell gel beads in the emulsion according to the manufacturer’s protocol. Captured cells were lysed and the released RNA were barcoded through reverse transcription in individual GEMs. Cell-barcoded 3′ gene expression libraries were sequenced on an Illumina NovaSeq6000 system by Shanghai Biochip Co., Ltd., Shanghai, China.

### Data processing

Raw sequencing data was processed by Cell Ranger (10xGenomics, version cellranger-6.0.0) pipeline and aligned to the human reference genome (GRCh38) to generate Gene-Barcode matrices of gene expression. The Gene-Barcode matrices containing the barcoded cells and gene expression counts were imported into the Seurat R toolkit (R version 4.0.3 (2020–10–10)). Cells with gene number (<300 & > 6000) or high mitochondrial transcript ratio (<25%), and genes expressed in less than 3 cells were all excluded. After removing unwanted cells from the dataset, all samples were combined with function “merge”. Next, we employed a global-scaling normalization method “LogNormalize” to normalize the feature expression measurements (UMI counts) for each cell by the total expression and the canonical correlation analysis (CCA) method to remove the batch effect.

### scRNA-seq clustering and cell type annotation

Highly variable genes (top 4000) were extracted to perform the principal component analysis (PCA) and top 30 of significant principal components were used for cluster analysis. Clusters were visualized using the Uniform Manifold Approximation and Projection (UMAP) and t-Distributed Stochastic Neighbor Embedding (t-SNE). Marker genes for each cluster, cell type and subgroup were identified by contrasting gene expression of cells from certain cluster, cell type or subgroup to that of others using the Seurat FindMarkers function. The types of cell clusters were annotated by specific marker genes of published cell types.

### Enrichment analysis

GO enrichment, KEGG enrichment of markers from cluster or cell type were performed using clusterProfiler software with Benjamini-Hochberg multiple testing adjustment, using marker genes from Seurat software with wilcox test and log-scale foldchange (logfc.threshold >= 0.25). The results were visualized using R package.

### Gene Set Enrichment Analysis (GSEA) analysis

GSEA was performed by using clusterProfiler software, which used predefined gene sets from KEGG Pathway Database. The genes in selected gene sets from distinct cell types of four samples were used. Gene expression data was calculated by mean umi count of genes in one cell type and the rest cell types, respectively.

### SingleR analysis for annotation of blood cells

Cell types of blood cell and stromal cell subclusters were annotated by SingleR (1.4.1). Unbiased cell type recognition from single-cell RNA sequencing data was performed by leveraging reference transcriptomic datasets of pure cell types to infer the origin of each single cell independently. For human, (HumanPrimaryCellAtlasData) was used.

### Single cell velocity analysis

To obtain the cell trajectories of stromal cells, the scVelo (0.2.0) package with default parameter was applied for studying the cellular differentiation status based on the normalized cell matrix from selected cells to solve the transcriptional dynamics of splicing kinetics using a likelihood-based dynamical model.

### Cell communication analysis

To enable a systematic analysis of cell-cell communication molecules, cell communication analysis based on the CellPhoneDB, a public repository of ligands, receptors, and their interactions, was applied. Significant mean and cell communication significance (p-value<0.05) was calculated based on the interaction and the normalized cell matrix achieved by Seurat Normalization. To identify potential interactions between and within immune cells and stromal cells, we used CellPhoneDB 2.0 with parameters threshold =0.25 and iterations=1000, which contains a curated repository of ligand-receptor interactions and a statistical framework for inferring lineage-specific interactions.

### Immunohistochemical analysis in the human NP samples

Samples were sectioned at a thickness of 5 µm. All the sections were incubated for 1 hour with primary antibody directed against IL1B (Cell Signaling Technology, #2022, 1:200), COL2A1 (Santa Cruz, sc-52658, 1:200), COL4A1 (Thermo Fisher Scientific, PA5-85634, 1:500), LGALS1 (Cell Signaling Technology, #5418, 1:200), MMP3 (Abcam, ab223666, 1:500) after blocking endogenous peroxidase using 3% hydrogen peroxide for 5 minutes at room temperature. After rinsing, the sections were incubated for 1 hour with biotinylated horseradish peroxidase-conjugated goat antirabbit IgG. Diaminobenzidine was used to develop peroxidase staining.

### Extraction, culture and identification of NPCs

NP samples were placed in a 37° incubator for digestion for a half hour using 0.25% trypsin. After centrifugation at 1500 RPM/min, the supernatant was removed, 0.1% type II collagenase (Thermo Fisher Scientific, 17101015) was added, and the cells were collected in an incubator at 37°C overnight. Cultured cells were validated by immunofluorescence staining with aggrecan (Abcam, ab36861, 1:500), which was NP cell-specific markers (**Supplementary Figure S1**).

### Immunofluorescence staining *in vitro*

After treated by the recombinant proteins such as TNF-α (MedChemExpress, HY-P7058), CD74 (MedChemExpress, HY-P72931) and CCL-3 (MedChemExpress, HY-P7256) for 24 hours, NP cells were further processed for immunofluorescence staining detection of the ossification markers including COL1A1, and RUNX2. Twenty thousand living cells were loaded onto each coverslip and incubated overnight. PFA (4%) was fixed at room temperature for 15 min. Then, blocking solution (containing 10% horse serum, 1% BSA and 0.1% Triton X-100 DPBS) was added and blocked for 1 hour. The blocking solution was discarded, and diluted primary antibodies including COL1A1 (ABclonal, A1352, 1:100), and RUNX2 (Thermo Fisher Scientific, 41-1400, 1:200) were added for incubation. After rinsing, the coverslips were placed in the dark and incubated in florescent-labeled goat-anti-rabbit IgG for 1 hour. Then, prolong gold antifade reagent with DAPI was added to the coverslips. All coverslips were randomly photographed, and at least 200 cells were counted for analysis (Leica DMI 6000B).

### Establishment of the rat disc degeneration model and validation by X-ray, MRI imaging, HE staining and S-O staining

Research protocols involving animals were approved by the Ethics Committee of Shanghai Jiaotong University First People’s Hospital (2021AWS0022). Twenty-four rats’ coccyx 6-7 intervertebral discs were punctured by a 26-G syringe needle to establish the degeneration group (annulus fibrous puncture group: AFP group) (11). For twelve rats, the TNF-α specific inhibitor (MedChemExpress, Infliximab: HY-P9970) were injected during the puncture process. After 4 weeks, all the rats were anesthetized for the further analysis. X-rays and MRI were performed to determine the degeneration degree in different groups. Moreover, the rats were sacrificed at 12 weeks after puncture. X-rays and MRI were performed again to evaluate the degeneration of rat intervertebral disc. Additionally, HE staining and safranin O-fast green staining were further used to validate the degeneration of rat intervertebral disc. Moreover, we also detected the expression of TNF-α (Cell Signaling Technology, #3707, 1:500), Col-1 (Abcam, ab34710, 1:200), Runx-2 (Abcam, ab192256, 1:200) *in vivo* after harvesting disc tissues using immunohistochemical analysis.

### Statistical analysis

SPSS V.18.0 software was utilized for statistical analysis. The measurement data were expressed as mean ± standard deviation. Differences between two groups were analyzed using two-tailed Student’s t-test. One-way analysis of variance was employed to compare all parameters among multiple groups, and LSD tests were performed for pairwise comparisons within groups. *P*<0.05 was considered statistically significant.

## Results

### Comprehensive scRNA-seq analysis of human degenerative NP identified five distinct cell populations

**Figure 1:**
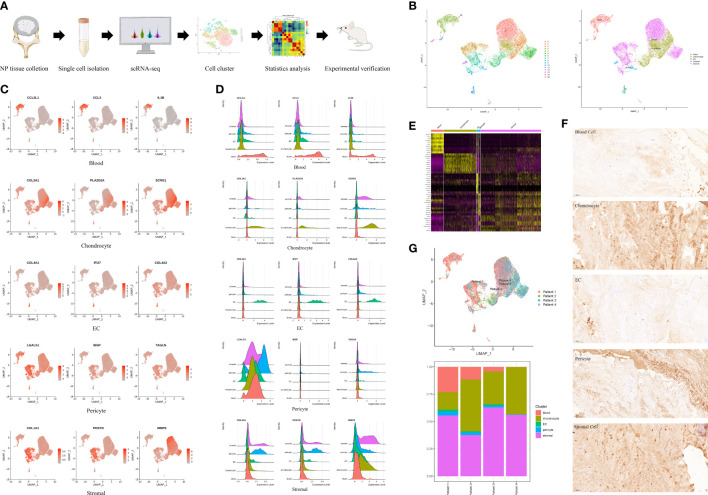
A single cell RNA-seq (scRNA-seq) of human degenerative nucleus pulposus (NP). **(A)** Schematic workflow of the experimental strategy. **(B)** Sixteen clusters (0-15) of human degenerative NP were identified by specifical genes into five cell clusters and shown by uniform manifold approximation and projection (UMAP). **(C)** Feature plot showing differentially expressed genes in five clusters. **(D)** Ridge plot showing differentially expressed genes in five clusters. **(E)** Heatmap showing differentially expressed genes in five clusters. **(F)** Representative immunohistochemistry staining of IL1B, COL2A1, COL4A1, LGALS1, COL1A1, for the five cell clusters of degenerative human NP. Scale bar, 100 μm. n=6. **(G)** UMAP indicating the heterogeneity of cell cluster percentage in four samples.

We dissociated NP tissues from four IDD patients with different degrees of degeneration and performed scRNA-seq (**Figure 1A**, **Table 1**). After quality control, 18,855 nuclear pulposus cells (NPCs) with a median of 1053 genes per cell remained and were clustered into sixteen populations. Immune cell marker genes (CCL3L1, CCL3, and IL1B) were ubiquitously expressed in the blood clusters (Clusters 4, 11, 13, 14, and 15) (12). The specific genes (COL2A1, PLA2G2A, and SCRG1) of in Clusters 1, 3, and 6 were chondrocyte marker genes (13). COL4A1, IFI27, and COL4A2 were the most highly expressed in endothelial cells (ECs) (Cluster 10) (14). Pericytes (Clusters 9 and 12) were identified by marker gene expression (LGALS1, IBSP, and TAGLN) (15). Stromal cells (Clusters 0, 2, 5, 7, and 8) were mainly identified by the expression of genes including COL1A1, POSTN, and MMP3 (**Figure 1B**) (16). These marker genes are shown in feature plots, ridge plots and heatmaps (**Figures 1C–E**). Immunohistochemistry validated the expression of specific markers for each NP cell cluster at the protein level (**Figure 1F**). Interestingly, the UMAP shows the heterogeneity among these samples; therefore, the proportions of different cell clusters were calculated among the four patients. We found no pericytes or blood cells in Patient 4, which was probably associated with a reduction in vessel ingrowth in degenerative NP due to the young age and short course of disease in this patient (**Figure 1G**) (17). Overall, these results revealed the atlas of human NP during IDD pathogenesis at the single-cell level.

**Table 1:** IDD patients characteristics.

Patient ID	Patient 1	Patient 2	Patient 3	Patient 4
Age (years)	20	69	23	28
Gender	Female	Female	Male	Female
Weight (Kg)	58	52	23	28
Pfirrmann Grading	II	III	III	II
CRP (mg/dL)	0.6	1.0	0.4	3.1
WBC (10^9^/L)	5.94	6.57	6.33	4.62
Lymphocyte (10^9^/L)	1.52	2.27	1.59	0.75
Monocyte (10^9^/L)	0.29	0.33	0.39	0.34
Blood Glucose (mmol/L)	3.97	5.21	5.2	5.19
ESR (mm/L)	2	10	3	5
ALT (U/L)	19.0	20.3	33.0	13.6
AST (U/L)	15.75	19.68	31.0	15.65
Total Protein (g/L)	69.81	68.43	78.3	59.83
Serum Albumin (g/L)	44.1	39.0	46.7	37.6
BUN (mmol/L)	7.6	4.8	6.16	4.7
Cre (umol/L)	51.0	61.6	80.5	64.5
TT (s)	17.6	18.1	17.1	16.5
APTT (s)	27.1	27.1	26.3	25.3

### Enrichment analysis revealed activation of the immune response in blood cells associated with IDD pathogenesis

**Figure 2:**
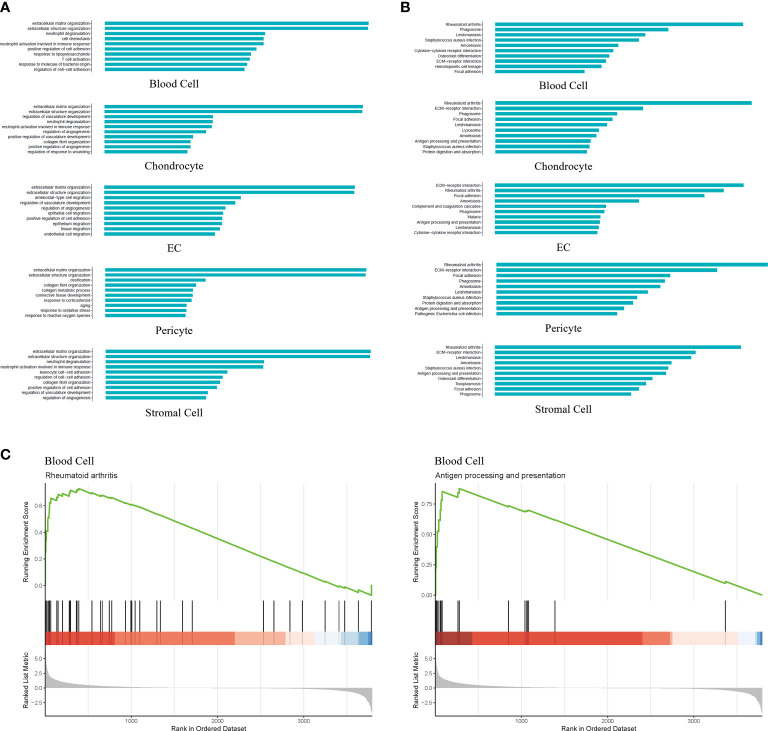
GO, KEGG and Gene Set Enrichment Analysis (GSEA) revealed the enrichment of biological processes and pathways in different cell types. **(A)** Bar plot indicating top 10 biological processes enriched in five cell types. **(B)** Bar plot depicting top 10 signaling pathways enriched in five cell types. **(C)** GSEA showing signaling pathways including Rheumatoid arthritis and Antigen processing and presentation were significantly activated in the blood cell cluster.

To investigate the potential functions of the differentially expressed genes (DEGs) identified in the five cell types, we used the Gene Ontology (GO) and Kyoto Encyclopedia of Genes and Genomes (KEGG) databases to explore the biological processes and pathways, respectively. The DEGs of blood cells were mainly enriched in processes related to extracellular matrix organization, cell chemotaxis and the immune response. The DEGs of chondrocytes were enriched in extracellular matrix organization, regulation of vasculature development and immune responses. The DEGs of ECs were enriched in extracellular matrix organization, the regulation of angiogenesis and cell migration. The DEGs of pericytes were enriched in extracellular matrix organization, ossification and aging. The DEGs of stromal cells were enriched in extracellular matrix organization, immune response, and cell adhesion (**Figure 2A**). Comprehensive analysis showed that the five cell types in degenerative NP were mainly enriched in extracellular matrix organization, the immune response, and ossification. Pathway analysis showed that the DEGs of blood cells were enriched in rheumatoid arthritis, phagosomes, and osteoclast differentiation. The DEGs of chondrocytes were enriched in rheumatoid arthritis, phagosome, and antigen processing and presentation. The DEGs of ECs were enriched in ECM-receptor interactions, rheumatoid arthritis, and antigen processing and presentation. The DEGs of pericytes were enriched in rheumatoid arthritis, ECM-receptor interaction, antigen processing and presentation. The DEGs of stromal cells were enriched in rheumatoid arthritis, ECM-receptor interactions, and osteoclast differentiation (**Figure 2B**). Comprehensive analysis showed that the five cell types in degenerative NP were mainly enriched in rheumatoid arthritis, antigen processing and presentation, and the ECM-receptor interaction pathway. As healthy intervertebral discs are immune-silenced, the appearance of blood cells in degenerative NP is an important event associated with IDD (5). Therefore, the activated status of pathways, including rheumatoid arthritis signaling and antigen processing and presentation signaling, in the blood cell cluster was further examined by gene set enrichment analysis (GSEA), which is an unbiased analysis that is independent of differentially expressed genes. The results showed that rheumatoid arthritis signaling and antigen processing and presentation signaling were activated in the blood cell cluster (**Figure 2C**). These results indicated that the immune response was activated in degenerative NP and may be associated with ECM remodeling, further inducing IDD.

### Subpopulation analysis indicated the infiltration of immune cells in degenerative NP

**Figure 3:**
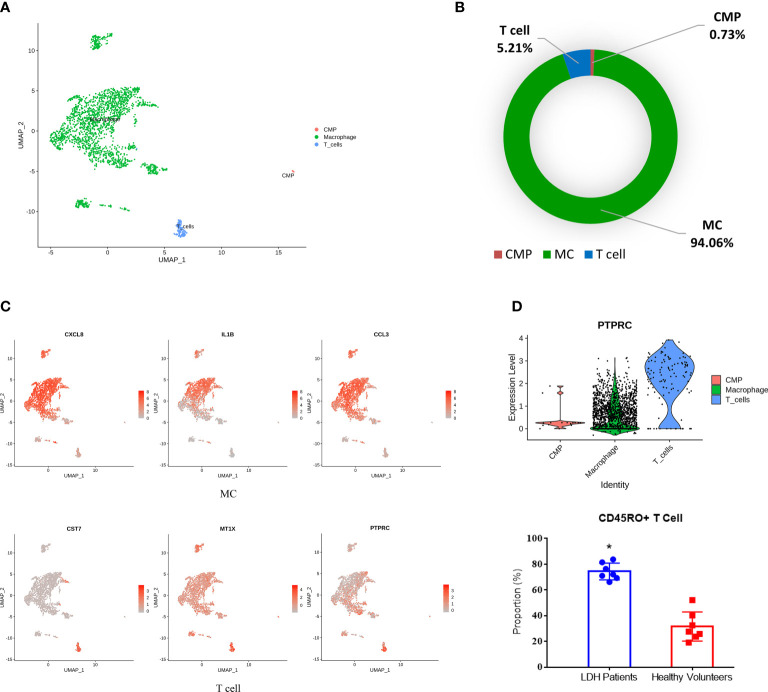
Identification of blood cell subpopulations and gene signatures during human IDD. **(A)** Three subpopulations of blood cells shown by UMAP. **(B)** Percentages of each subpopulation accounted in the whole blood cells. **(C)** Feature plot showing three highly expressed genes in macrophages and T cells. **(D)** The significant expression of PTPRC gene (encoding CD45RO protein) in T cells with the increase of CD45RO positive cells in blood samples of LDH patients. LDH, Lumbar disc herniation. *p<0.05.

To further investigate the effect of the immune response on IDD pathogenesis, we performed blood cell subpopulation analysis and revealed that blood cells could be further divided into MCs (CXCL8, IL-1B, and CCL3) (18), T cells (PTPRC, MT1X and CST7) (19) and CMPs (TPSB2, PRG2, TPSAB1) (**Figures 3A, C**). Statistical analysis of the proportion of blood cell subpopulations showed that the percentage of MCs was the highest, which was consistent with previous studies (**Figure 3B**) (20). Since CMPs accounted for 0.73% of all cells, we mainly focused on analyzing MCs and T cells. Interestingly, T cells were not located in normal NP, and this analysis showed the accumulation of T cells in degenerative NP; thus, we further examined blood samples from IDD patients. CD45, which is encoded by the PTPRC gene, is primarily expressed on the surface of T and B lymphocytes. Immunoassays showed that the proportion of CD45RO+ T cells (associated with activated and differentiated lymphocyte phenotypes) was significantly increased in the blood of LDH patients compared with that of healthy volunteers (**Figure 3D**), which indicated that CD45RO+ T cells may be an immunotherapy target in the future.

### Subpopulations (3, 4, 5) of stromal cells associated with NP ossification and extracellular matrix organization in degenerative NP

**Figure 4:**
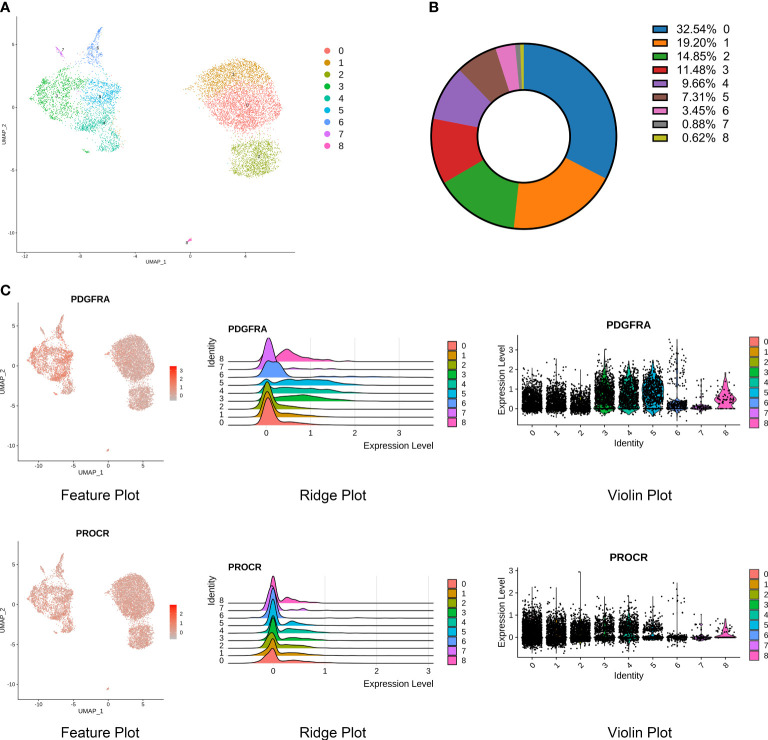
Identification of stromal cell subpopulations and gene signatures during human IDD. **(A)** Nine subpopulations of stromal cells shown by UMAP. **(B)** Percentages of each subpopulation accounted in the whole stromal cells. **(C)** High expression of PDGFRA and PROCR, signatures of progenitor cells, shown by feature, ridge, and violin plots.

**Figure 5:**
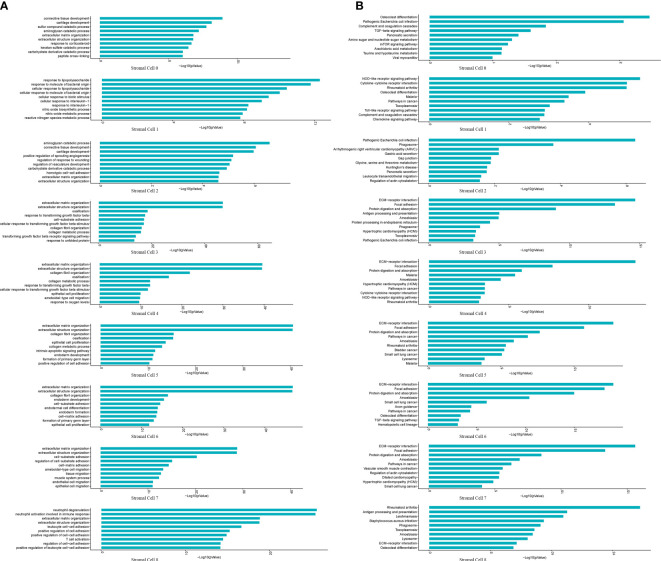
GO and KEGG revealed the enrichment of biological processes and pathways in stromal cell subpopulations. **(A)** Bar plot indicating top 10 biological processes enriched in the nine subpopulations of stromal cells. **(B)** Bar plot depicting top 10 signaling pathways enriched in the nine subpopulations of stromal cells.

Stromal cells are the progenitor cells in the NP region and can contribute to regeneration of the adult intervertebral disc. Additionally, this cell type accounted for a high proportion in the scRNA-seq results. Therefore, we further classified stromal cells into 0-8 subpopulations based on their percentages (**Figures 4A, B**). PDGFRA and PROCR were universally expressed in all subpopulations, similar to the results of a previous study (**Figure 4C**) (3). We further analyzed the biological process and pathway enrichment in the subpopulations of stromal cells. The DEGs of Clusters 0 and 2 were enriched in connective tissue development, cartilage development, and extracellular matrix organization. The DEGs of Cluster 1 were enriched in the response to lipopolysaccharide, bacterial origin and interleukin-1. The DEGs of Clusters 3, 4 and 5 were enriched in extracellular matrix organization, ossification and collagen metabolic processes. The DEGs of Clusters 6 and 7 were enriched in extracellular matrix organization, endodermal cell differentiation, and cell−matrix adhesion. The DEGs of Cluster 8 were enriched in neutrophil degranulation, neutrophil activation involved in the immune response, and extracellular matrix organization (**Figure 5A**). Pathway analysis showed that the DEGs of Clusters 0 and 2 were enriched in osteoclast differentiation, pathogenic *Escherichia coli* infection, complement and coagulation cascades and phagosomes. The DEGs of Cluster 1 were enriched in cytokine−cytokine receptor interaction, rheumatoid arthritis and osteoclast differentiation. The DEGs of Clusters 3, 4, 5, 6, and 7 were enriched in ECM-receptor interactions, focal adhesions, protein digestion and absorption. The DEGs of Cluster 8 were enriched in rheumatoid arthritis, antigen processing and presentation, ECM-receptor interactions and osteoclast differentiation (**Figure 5B**). These results indicated that the functions of each subpopulation were different and that Subpopulations 3, 4, and 5 were mainly associated with NP ossification and extracellular matrix organization in degenerative NP. Additionally, some novel markers were identified in the subpopulations of stromal cells (**Supplemental Figure S2**). These findings provide references for investigating the role of stromal cell subpopulations in the NP.

### RNA velocity analysis revealed that stromal cells differentiated toward an ossification phenotype

**Figure 6:**
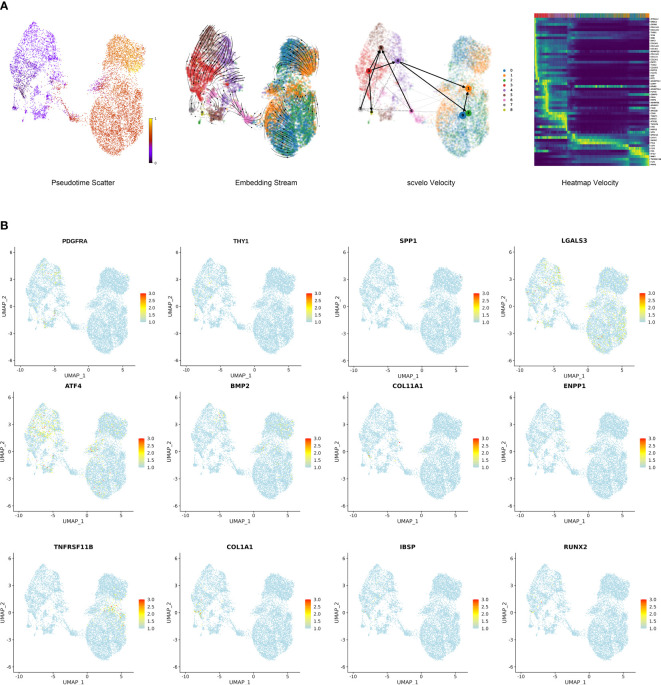
Single-cell trajectory analysis of stromal cells in degenerative NP. **(A)** Trajectory of nine cell types with differentially expressed genes showed by pseudotime_scatter, embedding_stream and scvelo velocity. Heatmap velocity indicating the specific genes at the differentiation stages of stromal cells. **(B)** Feature plot showing the expression of several progenitor cell marker genes and ossification marker genes.

To further investigate the differentiation of stromal cells and corresponding gene expression, we conducted a velocity analysis of stromal cells. Subpopulation 3 was present at the start in the stromal cell differential trajectory (**Figure 6A**). Subpopulation 3 could differentiate into Subpopulation 5 and Subpopulation 8. Additionally, Subpopulation 3 could differentiate into Subpopulations 1, 2 and 4. These findings suggest that Subpopulation 3 possesses progenitor properties. The heatmap shows the specific genes at the differentiation stages of stromal cells. ZFP36L2, ADAM12, FHL1, FGF1, BTG2, FGF2, JARID2, LY6K, FOS, CDK6, ARID5B, GPRC5A, TWIST1, TCF4, GREM1, FOSB, CSF1, DKK3, SMOC1, and FGF18 were associated with cell proliferation, differentiation, development and regeneration. EFNB2, FAT1, ENAH, and POSTN were associated with cell connection, adhesion and motility. MMP2, MMP3, MMP10, MMP13, CEMIP, CEMIP2, COL3A1, COL5A1, COL5A2, COL6A3, COL12A1, COL14A1, COL15A1, TNFAIP6, ADAMTS12, ADAMTS1, ADAMTS5, ADAMTS14, THBS1, and THBS2 were associated with the extracellular matrix and extracellular matrix degradation (**Figure 6A**). Overall, we believed that stromal cells transferred from the characteristics of cell proliferation to the characteristics of cell adhesion and motility and then participated in the regulation of the extracellular matrix, which further resulted in the appearance of ossification characteristics. PDGFRA and THY1 (CD90) are signatures of progenitor cells that were notably expressed in Subpopulation 3; therefore, we further confirmed Subpopulation 3 as the progenitor cells in degenerative NP (**Figure 6B**). Importantly, ossification marker genes, such as OGN, CHSY1, ATP2B1, SNORC, ATF4, BMP2, COL11A1, ENPP1, SPP1, TNFRSF11B, COL1A1, IBSP, RUNX2, and LGALS3, were significantly expressed throughout the stromal cell differentiation process (**Figures 6A, B**) (21, 22). Activation of these ossification genes may promote stromal cell differentiation toward an ossification phenotype, further interfering with the repair functions of stromal cells.

### Cellphone DB analysis revealed that immune cells promoted the differentiation of stromal cells toward an ossification phenotype

**Figure 7:**
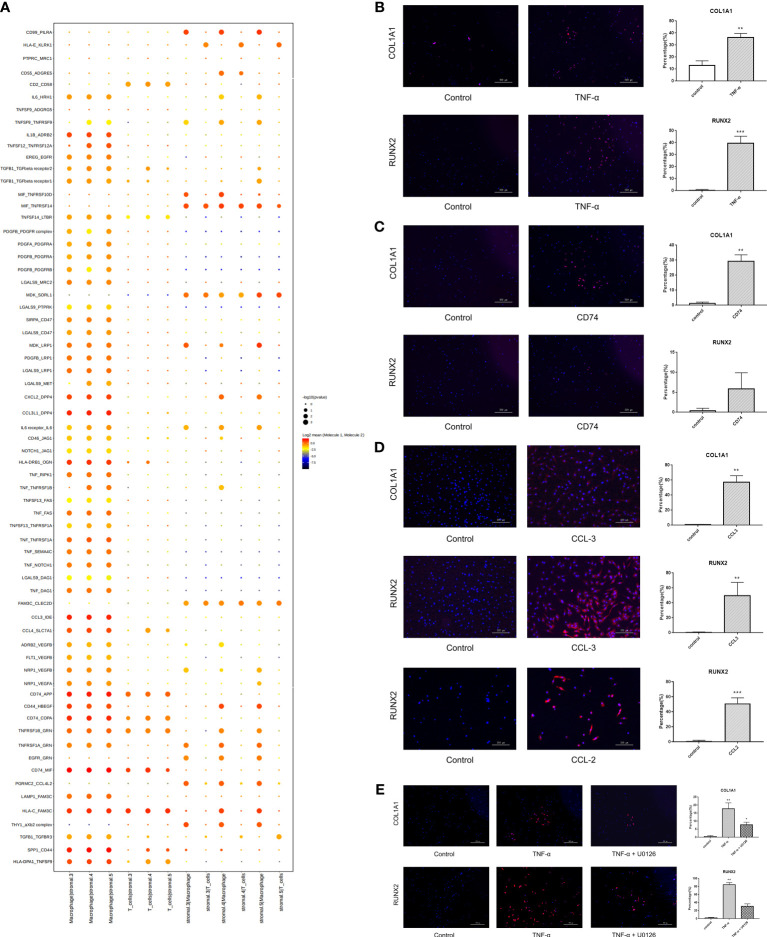
Cell-to-cell communication analysis revealing stromal cells differentiate towards ossification by interacting with immune cells. **(A)** Cellphone DB analysis showing many interactions between immune cells and stromal cells 3, 4, 5. **(B)** Human degenerative NP cells were treated with TNF-α for 24 hours to explore the expression of ossification markers (COL1A1 and RUNX2) by immunofluorescence staining. PBS was used as a negative control. Scale bar, 500 μm. n=5, * vs control, p<0.05. **(C)** Human degenerative NP cells were treated with CD74 to explore the expression of ossification markers (COL1A1 and RUNX2) by immunofluorescence staining. PBS was used as a negative control. Scale bar, 500 μm. n=5, * vs control, p<0.05. **(D)** Human degenerative NP cells were treated with CCL-3 and CCL-2 to explore the expression of ossification markers (COL1A1 and RUNX2) by immunofluorescence staining. PBS was used as a negative control. Scale bar, 250 μm. n=5, * vs control, p<0.05. **(E)** Human degenerative NP cells were treated with TNF-α and MAPK inhibitor (U0126) to explore the expression of ossification markers (COL1A1 and RUNX2) by immunofluorescence staining. PBS was used as a negative control. Scale bar, 500 μm. n=5, * vs control, p<0.05. ** p<0.01; *** p<0.001.

As shown in the stromal cell subpopulation analysis, Clusters 3, 4, and 5 were enriched in extracellular matrix organization, ossification and collagen metabolic processes. The differentiation of stromal cells may be affected by immune cells, and so Cellphone DB analysis was performed. The results showed that MCs and T cells had more interactions with Clusters 3, 4, and 5 than with the other clusters (**Supplementary File 1**). Therefore, we focused on the effect of immune cells (including macrophages and T cells) on these clusters. We found strong ligand−receptor interactions between MCs and T cells with stromal cells, including IL1B_ADRB2, CXCL2_DPP4, CCL3L1_DPP4, HLA-DRB1_OGN, TNF_RIPK1, TNF_TNFRSF1B, TNF_FAS, TNF_TNFRSF1A, TNF_SEMA4C, TNF_NOTCH1, TNF_DAG1, CCL3_IDE, CCL4_SLC7A1, CD74_APP, CD44_HBEGF, CD74_COPA, CD74_MIF, HLA-C_FAM3C, SPP1_CD44, and HLA-DAP1_TNFSF9. Further analysis revealed that these interactions were mainly associated with cell binding and adhesion, cell growth, and signal transduction and affected the biological processes of stromal cells, including ossification, cell proliferation and differentiation (**Figure 7A**, **Supplementary File 2**). Among these interactions, TNF-α, CCL-3 and CD74 frequently acted as ligands, suggesting that TNF-α, CCL-3 and CD74 may play key roles in inducing the ossification of stromal cells. Therefore, immunofluorescence analysis was further performed for validation *in vitro*. NP cells were isolated from human tissues and treated with recombinant TNF-α, CD74 and CCL-3 proteins, and the expression of ossification markers, including COL1A1 and RUNX2, was measured. The results showed that these factors successfully induced the expression of COL1A1 and RUNX2 (**Figures 7B–D**). As shown previously, TNF-α interacted with TNFRSF1B and TNFRSF1A, which are receptors of the MAPK signaling pathway. To further clarify the signaling pathways in stromal cell ossification that are induced by immune factors, we focused on the effect of TNF-α on the MAPK signaling pathway. The results showed that the upregulated expression of ossification genes (COL1A1 and RUNX2) was significantly reversed after the administration of a MAPK inhibitor (U0126: HY-12031A), which indicated that TNF-α could induce NP cell ossification by activating the MAPK signaling pathway (**Figure 7E**). Additionally, we found strong ligand−receptor interactions between stromal cells, MCs and T cells, including CD99_PILRA MIF_TNFRSF14, MDK_SORL1, FAM3C_CLEC2D, CD44_HBEGF, TNFRSF1B_GRN, TNFRSF1A _GRN, PGRMC2_CCL4L2, and the THY1_axb2 complex, which may change immune cell functions such as cell proliferation, apoptosis and differentiation.

### Inhibiting TNF-α *in vivo* alleviated intervertebral disc ossification and degeneration

**Figure 8:**
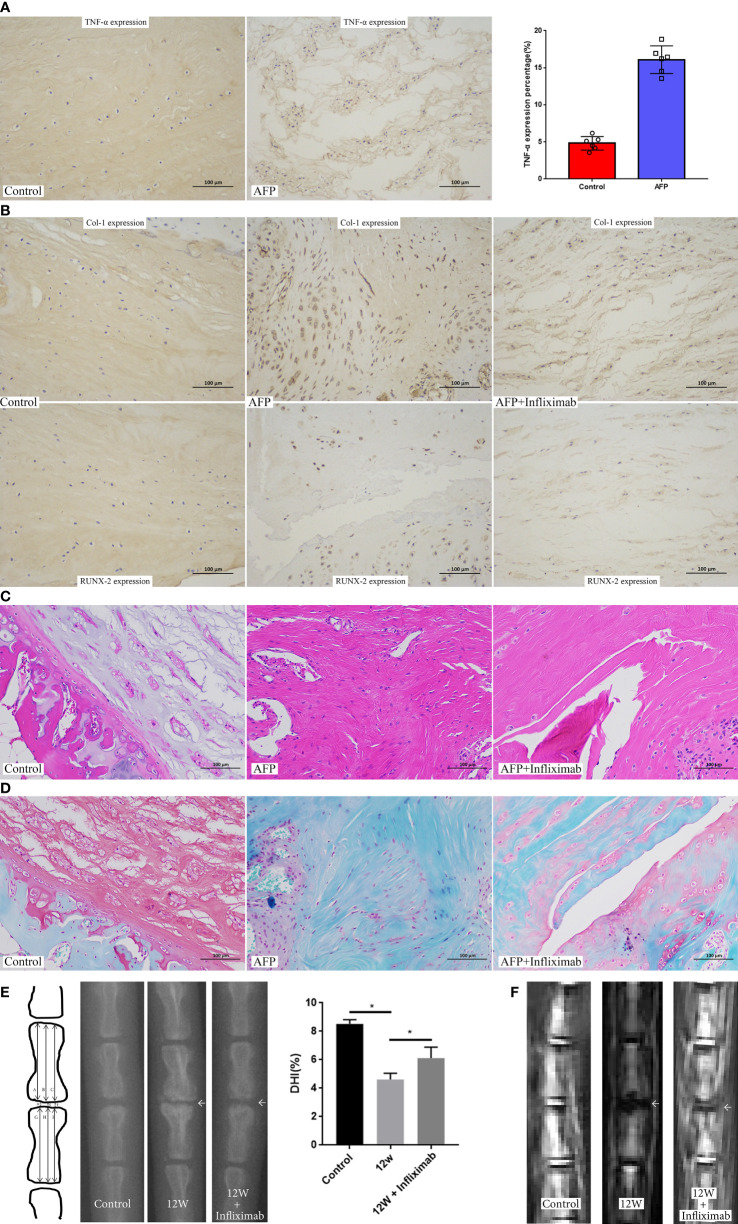
Inhibition of TNF-α in the established rat disc degeneration model. **(A)** The expression of TNF-α in the established rat disc degeneration model. **(B)**The expression of ossification markers (COL1A1 and RUNX2) after inhibiting the role of TNF-α by administration of infliximab. **(C)** HE staining of intervertebral disc after inhibiting the role of TNF-α by administration of infliximab. **(D)** Safranin O-fast green staining of intervertebral disc after inhibiting the role of TNF-α by administration of infliximab. **(E)** X rays of rat coccyx disc after inhibiting the role of TNF-α by administration of infliximab. **(F)** MRI imaging of rat coccyx disc after inhibiting the role of TNF-α by administration of infliximab. AFP, annulus fibrous puncture. *p<0.05.

Furthermore, we examined the role of inhibiting immune factors, such as TNF-α, on NP ossification and IDD using a rat coccyx disc degeneration model. First, narrowing of the intervertebral space was found by X-ray in the degeneration group (**Supplemental Figure S3A**). Additionally, MRI showed that the NP signal switched to gray with decreased disc height in the degeneration group (**Supplemental Figure S3B**). HE staining and safranin O-fast green staining indicated that the NP had a long spindle shape with a decrease in cell number and disordered collagen fibers. The boundary between the AF and NP became blurred in the degeneration group (**Supplemental Figures S3C, D**). These results demonstrated the successful establishment of the disc degeneration animal model. In this animal model, immunohistochemical staining showed significantly increased expression of TNF-α in the AFP group (**Figure 8A**). Blocking TNF-α with a specific inhibitor decreased the expression of ossification markers, including Col-1 and Runx-2 (**Figure 8B**), and improved disc morphology (**Figures 8C, D**). Furthermore, X-ray and MRI showed improvements in disc imaging (**Figures 8E, F**). Therefore, we clearly indicated that inhibiting TNF-α in the NP could alleviate intervertebral disc ossification and degeneration.

## Discussion

IDD is associated with important pathological changes, including phenotypic changes in NP cells and ECM degradation (23). Previous bulk RNA sequencing profiled NP cell phenotypic changes and matrix degradation (24); however, cell heterogeneity was lacking, and the markers were limited. Here, we determined cellular diversity with novel markers and signaling pathways involved in IDD pathogenesis at the single-cell level in human degenerative NP. Moreover, we demonstrated that immune cells that accumulated in degenerative NP could switch the differential trajectory of stromal cells, which are progenitor cells, and induce them to differentiate toward an ossification phenotype, further disturbing their capacity for endogenous repair.

Five cell populations were identified in this analysis. Chondrocytes are the most common cells in healthy NP and can synthesize proteoglycan and type II collagen (25); however, the number of chondrocytes was not highest in our study. Nutrient deficiency, nicotine and inflammatory factors in degenerative NP could inhibit chondrocyte proliferation and increase cell apoptosis to cause the decrease of chondrocytes (26). In chondrocytes, the classical gene Col2A1 and novel marker genes, including PLA2G2A and SCRG1, were expressed (27). PLA2G2A was significantly increased in samples from OA patients, which suggested that the increased expression of this gene in NP may be involved in NP degeneration. We identified stromal cells as the largest population and showed that these were progenitor cells that functioned in NP repair and regeneration. Among stromal cells, we found the expression of COL1A1, POSTN and MMP3, and COL1A1, which encodes type I collagen, was associated with NP degeneration (28). POSTN was significantly increased in the cartilage and bone of OA patients and was related to synovitis and the degeneration of articular cartilage (29). MMP3 is a key enzyme that degrades ECM, and inhibiting MMP3 can promote the expression of type II collagen and proteoglycan to delay NP degeneration (30).

The biological processes and pathways in each cell type were explored according to the differentially expressed genes. ECM organization is the key biological process associated with the maintenance of optimal NP function. During IDD, ECM organization is markedly altered and switches to degradation (31). Decreases in collagen-II and proteoglycan lead to intervertebral disc ossification; conversely, disc ossification can further cause NP cell death and reduce cell proliferation to exacerbate disc degeneration (32). Signaling pathway analysis suggested the activation of rheumatoid arthritis signaling and antigen processing and presentation signaling in the blood cell cluster in degenerative NP. Furthermore, we performed a deeper investigation of blood cells and stromal cells. We identified three clusters of blood cells: MCs, T cells, and CMPs. Nakazawa et al. revealed the infiltration of MCs in degenerative intervertebral discs by analyzing twelve human intervertebral disc specimens (33). Type II collagen and aggrecan protein in degenerative NP act as damage-associated molecular patterns (DAMPs), which activate macrophages to further secrete corresponding immune factors. Activated macrophages can further induce NP cells expressing Fasl to promote the expression of inflammatory mediators and ECM degradation enzymes, which accelerates NP degeneration (34). Similarly, Wang et al. indicated that Treg cells were significantly increased in degenerative NP and were associated with IDD and the occurrence of disc-derived lower back pain (35). Li showed that inhibiting CD8-positive T cells in a rat tail IDD model could delay NP degeneration (36). Interestingly, we found that CD45 RO+ T cells were increased in the NP tissue and blood of LDH patients. This finding was consistent with a study showing that CD4+CD45RO+ T cells (effector T cells) were significantly increased in the sequestered disc of lumbar disc herniation (37), which suggested that CD45RO+ T cells accumulated in degenerative NP and were important immune cells involved in IDD.

Endogenous progenitor cells function as reparative cells to maintain NP homeostasis and can delay NP degeneration. We identified nine clusters of stromal cells with well-defined progenitor marker genes, including PDGFRA and PROCR that were significantly increased in IDD patients and were related to the severity of disc degeneration. We also analyzed the biological process and pathway enrichment in the subpopulations of stromal cells, which indicated subpopulations (3–5) of stromal cells associated with NP ossification and extracellular matrix organization. Additionally, we studied the mechanism of NP ossification and measured the expression of ossification-related genes in stromal cells (OGN, CHSY1, ATP2B1, SNORC, ATF4, BMP2, COL11A1, ENPP1, SPP1, TNFRSF11B, COL1A1, IBSP, RUNX2, and LGALS3). ATF4, which is a novel transcriptional activator of Ihh, was expressed in proliferative growth plate chondrocytes and paces longitudinal bone growth. Knocking out the Atf4 gene decreased bone mass and delayed ossification (38). BMP2 is expressed in most degenerated discs and is associated with disc ossification (39). COL11A1, which was upregulated in MSCs in the growth plate and perichondrium, caused endochondral ossification (40). SPP1, which was significantly increased in degenerative IVD-MSCs, was related to bone matrix mineralization and ECM destruction (41). TNFRSF11B, which is known as osteoprotegerin (OPG), protects bone from excessive resorption by binding to RANKL and is positively correlated with IDD (42). COL1A1 is an important extracellular matrix component that promotes ossification. Mutations in this gene cause osteogenesis imperfecta, which is characterized by brittle bone fractures (43). IBSP, which is a key osteogenic factor, is differentially expressed in the nucleus pulposus, annulus fibrosus and articular chondrocytes (44). However, the role of IBSP in IDD has not yet been revealed and requires further study. Runx-2, which is expressed in degenerative intervertebral discs, induces osteocalcin expression to and is involved in IDD (21). The activation of these ossification-related genes in stromal cells may promote NP ossification.

Cellphone DB analysis indicated that both MCs and T cells could induce stromal cell differentiation toward an ossification phenotype through factors such as TNF-α, CCL-3, and CD74. TNF-α could enhance the bone-inducing capacity of BMP-2 and induce ectopic bone formation *in vivo* (45). As shown by KEGG analysis, the MAPK signaling pathway is activated in stromal cells, and this pathway is associated with cell growth, differentiation and ossification (46). Additionally, TNF-α is secreted by macrophages and can activate the classical MAPK signaling pathway (47). Therefore, we focused on the role of the MAPK signaling pathway in NP ossification in this study. The results showed that TNF-α could successfully activate the MAPK signaling pathway to induce NP ossification *in vitro*.

There are several limitations in this study. First, the sample size was relatively small, and more samples are needed for follow-up scRNA-seq studies. Second, to facilitate the analysis of such a large dataset, we categorized the differentially expressed genes by specific cell clusters. However, many marker genes were present in more than one cluster. The interactions between cell clusters containing these genes were not explored, leading to a simplified analysis. Third, the *in vitro* cell model that was used was NP cells but not stromal cells. Stromal cells need to be sorted for further validation in a future study. Fourth, the IDD rat model that was used in our study may differ from disc ossification animal models. Therefore, it is necessary to build a scientific disc ossification animal model to systematically study the mechanism.

**Figure 9:**
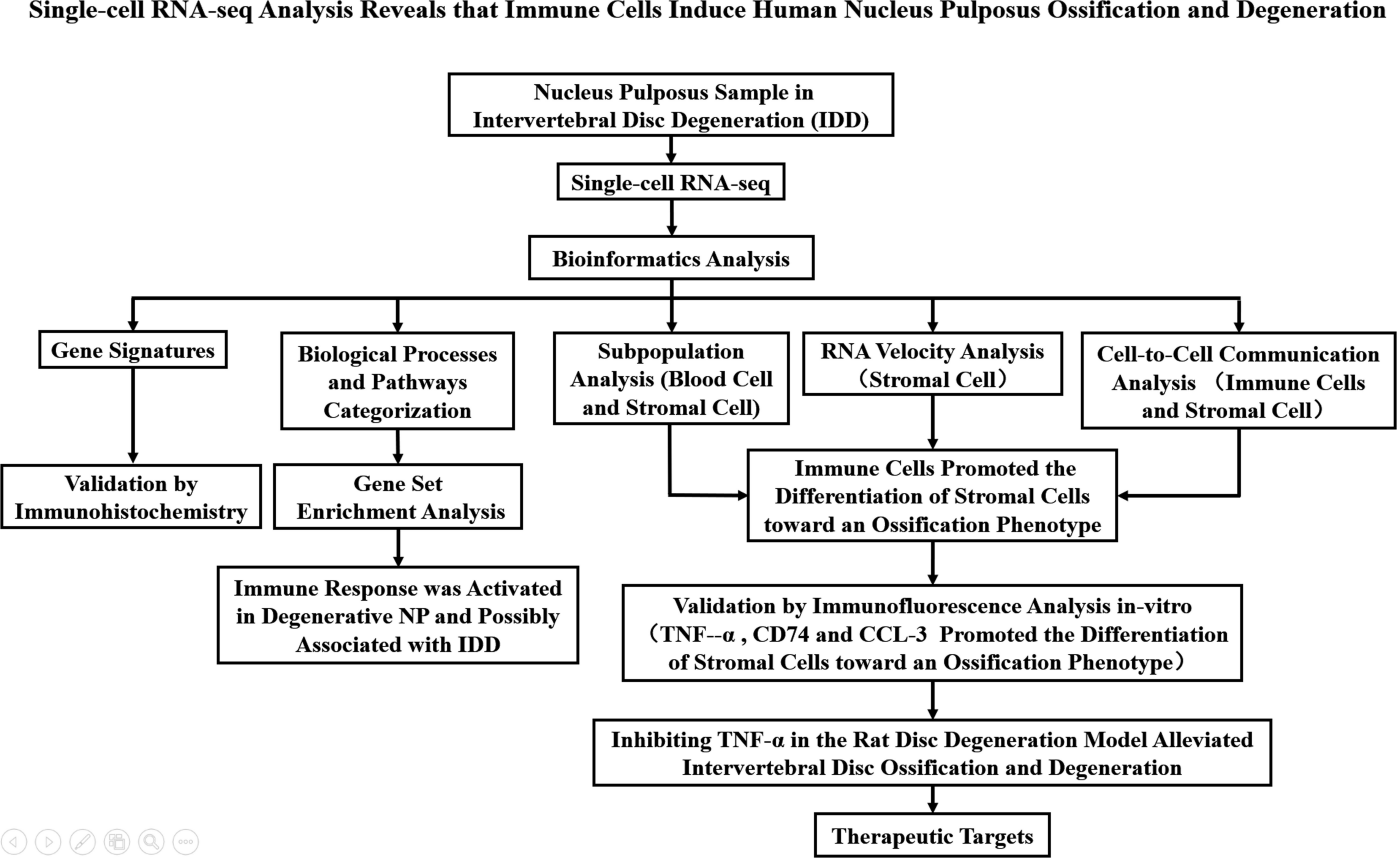
A flowchart that summarizes the overall thinking and method steps of the full text in the manuscript.

In summary, we performed transcriptomic profiling of degenerative NP at the single-cell level and showed an increase of macrophages and T cells in degenerative NP. Additionally, we identified stromal cells as progenitor cells with specific marker genes and determined the differentiation trajectory of stromal cells. We further showed activation of the immune response and ossification signaling pathways and showed that TNF-α affected stromal cell ossification by activating the MAPK signaling pathway. Inhibiting TNF-α in NP alleviated the ossification of NP, thus providing a therapeutic target to delay IDD (**Figure 9**).

## Data availability statement

The datasets presented in this study can be found in online repositories. The names of the repository/repositories and accession number(s) can be found below: GSE233666 (GEO).

## Ethics statement

The studies involving human participants were reviewed and approved by the Ethics Committee of Shanghai General Hospital, Shanghai Jiao Tong University School of Medicine. The patients/participants provided their written informed consent to participate in this study. The animal study was reviewed and approved by the Ethics Committee of Shanghai General Hospital, Shanghai Jiao Tong University School of Medicine.

## Author contributions

SG performed most of the experiments, analyses and writing; MY performed data accusation, writing and prepared the figures and tables; XL provided the input in writing the paper; ZL designed the outline and coordinated the writing of the paper; KL contributed to animal management; PL performed data analysis and writing; YL contributed to the writing and manuscript review; QF contributed to the conception of the study and manuscript review. SZ performed data analysis and manuscript review. GS performed data accusation and manuscript review. All authors contributed to the article and approved the submitted version.
