# Supplementary material for: Single-cell RNA-seq analysis reveals that immune cells induce human nucleus pulposus ossification and degeneration
Source: Front Immunol. 2023 Aug 10;14:1224627. doi: 10.3389/fimmu.2023.1224627 (PMC10449260; doi:10.3389/fimmu.2023.1224627)
Supplement: Supplementary file 2 [file DataSheet_2.pdf]

Gene pairs	Effects
IL1B_ADRB2	Mediates the catecholamine-induced activation of adenylate cyclase through the action of G proteins
CXCL2_DPP4	Regulates lymphocyte-epithelial cell adhesion, pericellular proteolysis of the extracellular matrix (ECM), the migration and invasion of endothelial cells into the ECM, promotion of lymphatic endothelial cells adhesion, migration and tube formation
CCL3L1_DPP4	Regulates lymphocyte-epithelial cell adhesion, pericellular proteolysis of the extracellular matrix (ECM), the migration and invasion of endothelial cells into the ECM, promotion of lymphatic endothelial cells adhesion, migration and tube formation
HLA-DRB1_OGN	Induces bone formation in conjunction with TGF-beta-1 or TGF-beta-2
TNF_RIPK1	Regulates cell death and kinase-independent scaffold functions regulating inflammatory signaling and cell survival, regulate necroptosis and apoptosis, involved in inflammatory response by promoting transcriptional production of pro-inflammatory cytokines, such as interleukin-6
TNF_TNFRSF1B	Regulates TNF-alpha function by antagonizing its biological activity to block TNF-alpha-induced apoptosis
TNF_FAS	Catalyzes the de novo biosynthesis of long-chain saturated fatty acids starting from acetyl-CoA and malonyl-CoA in the presence of NADPH.
TNF_TNFRSF1A	Contributes to the induction of non-cytocidal TNF effects including anti-viral state and activation of the acid sphingomyelinase.
TNF_SEMA4C	Plays an important role in cell-cell signaling, required for normal brain development, axon guidance and cell migration (By similarity), play a role in myogenic differentiation through activation of the stress-activated MAPK cascade
TNF_NOTCH1	Regulate cell-fate determination, affects the implementation of differentiation, proliferation and apoptotic programs. Involved in angiogenesis; negatively regulates endothelial cell proliferation and migration and angiogenic sprouting. Involved in the maturation of both CD4+ and CD8+ cells in the thymus.
TNF_DAG1	Regulates laminin and basement membrane assembly, extracellular matrix, cell survival, peripheral nerve myelination, nodal structure, cell migration.
CCL3_IDE	Regulates degradation of insulin, glucagon and other polypeptides.
CCL4_SLC7A1	Regulates the transport of the cationic amino acids (arginine, lysine and ornithine) in non-hepatic tissues
CD74_APP	Regulates neurite growth, neuronal adhesion and axonogenesis.
CD44_HBEGF	Regulates macrophage-mediated cellular proliferation.
CD74_COPA	Mediates biosynthetic protein transport from the ER via the Golgi up to the trans Golgi network.
CD74 _MIF	Regulates the function of macrophages in host defense
HLA-C_FAM3C	Promotes epithelial to mesenchymal transition.
SPP1_CD44	Regulates cell-cell interactions, cell adhesion and migration, extracellular matrix components, helping them to sense and respond to changes in the tissue microenvironment
HLA-DAP1_TNFSF9	Induces the proliferation of activated peripheral blood T-cells, regulate interactions between T-cells and B-cells/macrophages
