# Supplementary figures and images for: Single-cell RNA-seq analysis reveals that immune cells induce human nucleus pulposus ossification and degeneration
Source: Front Immunol. 2023 Aug 10;14:1224627. doi: 10.3389/fimmu.2023.1224627 (PMC10449260; doi:10.3389/fimmu.2023.1224627)

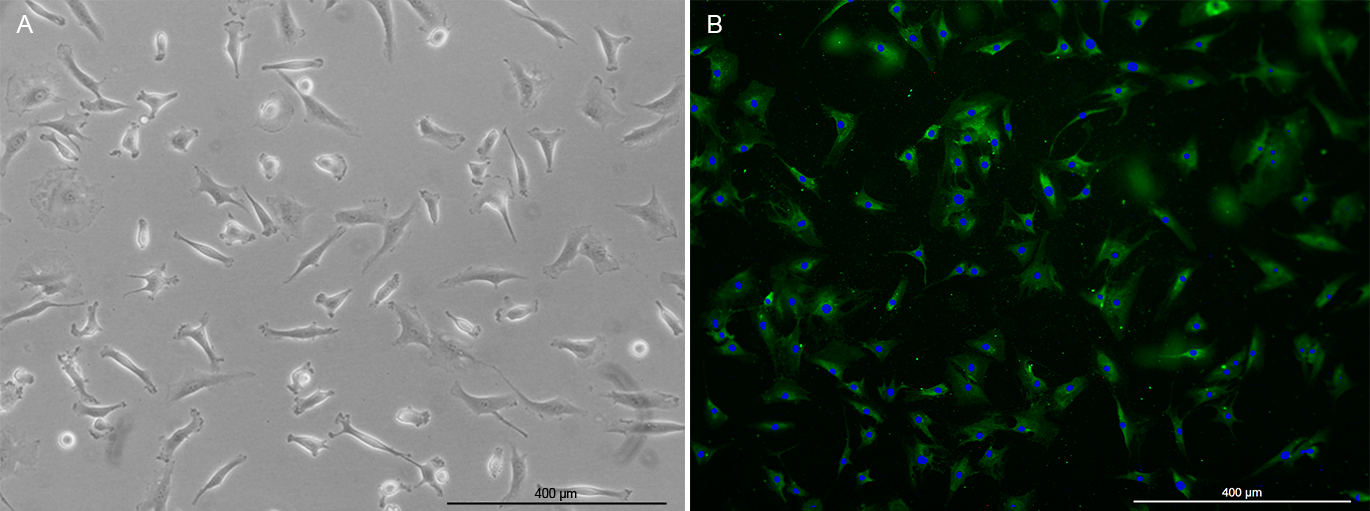

Supplement: Supplementary file 3 [file Image_1.tif]

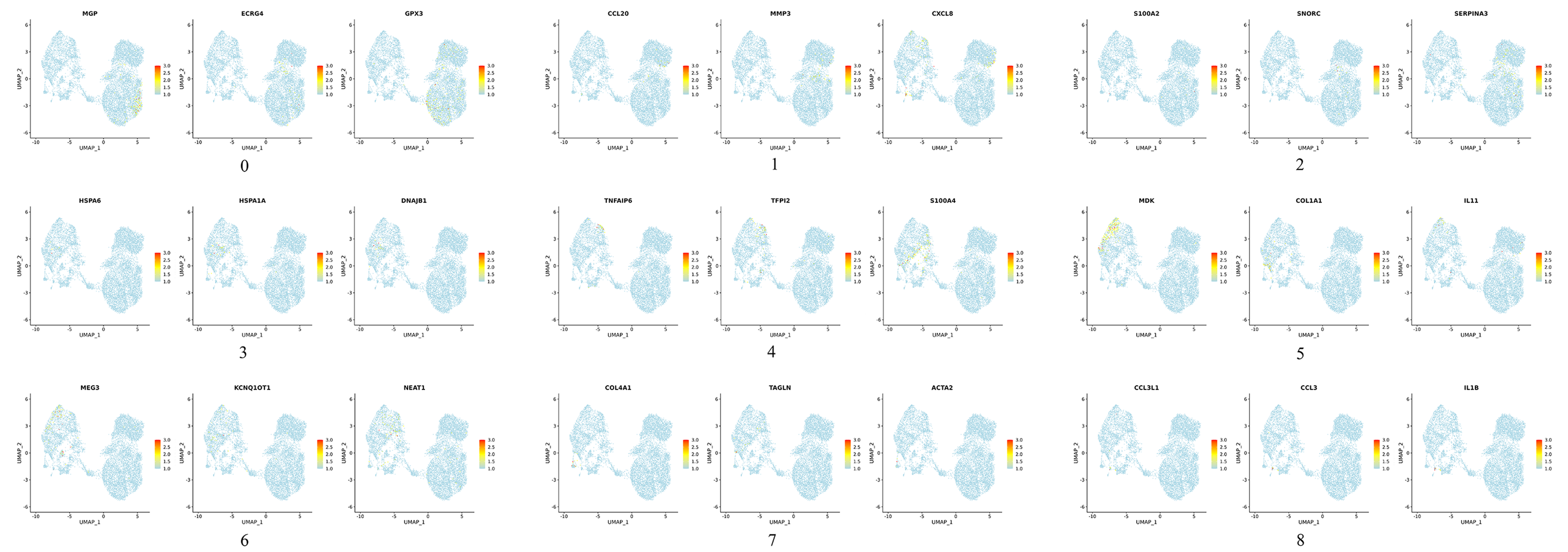

Supplement: Supplementary file 4 [file Image_2.tif]

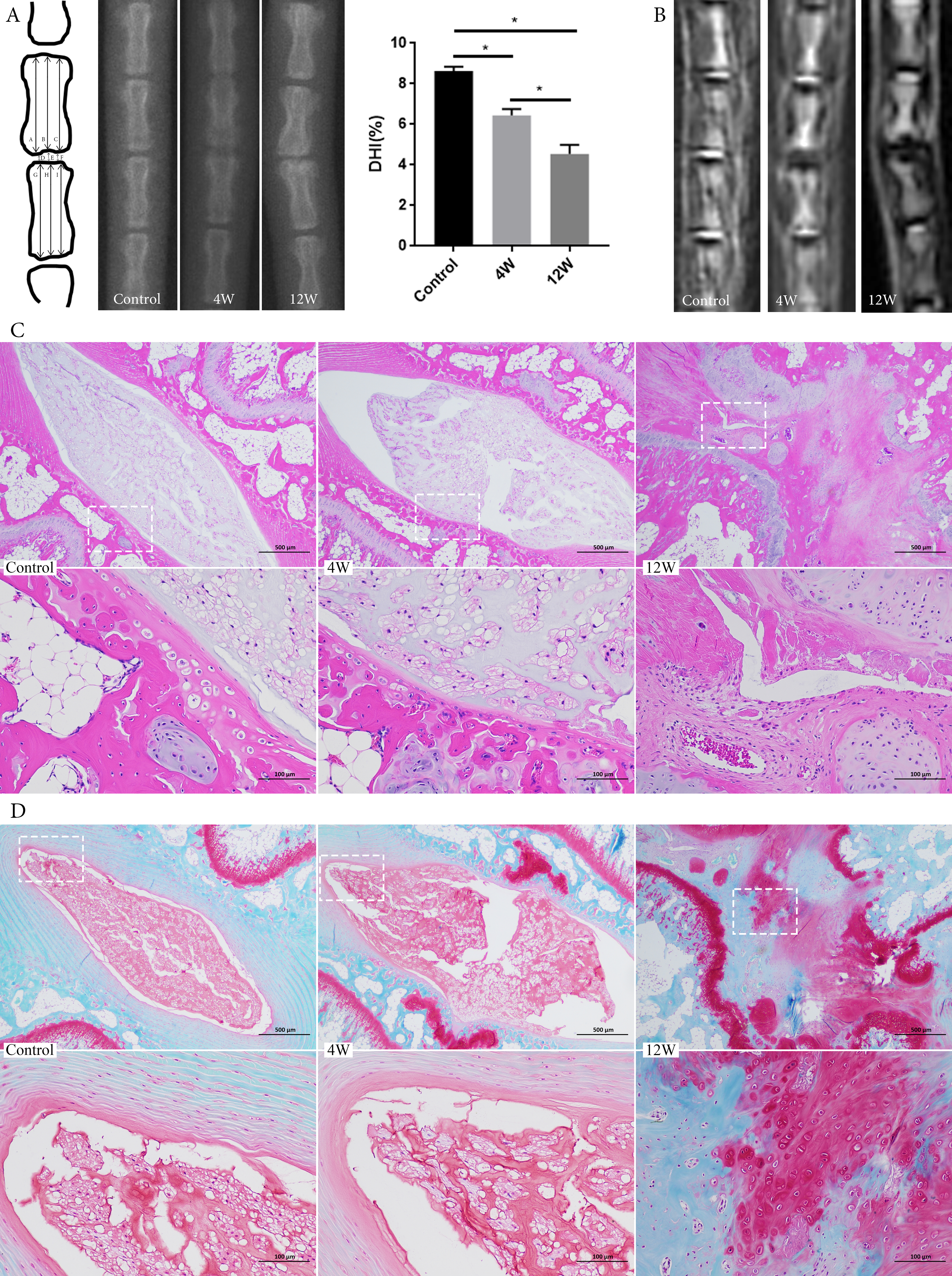

Supplement: Supplementary file 5 [file Image_3.tif]
