# Supplementary material for: Single-cell RNA-seq analysis reveals that immune cells induce human nucleus pulposus ossification and degeneration
Source: Front Immunol. 2023 Aug 10;14:1224627. doi: 10.3389/fimmu.2023.1224627 (PMC10449260; doi:10.3389/fimmu.2023.1224627)

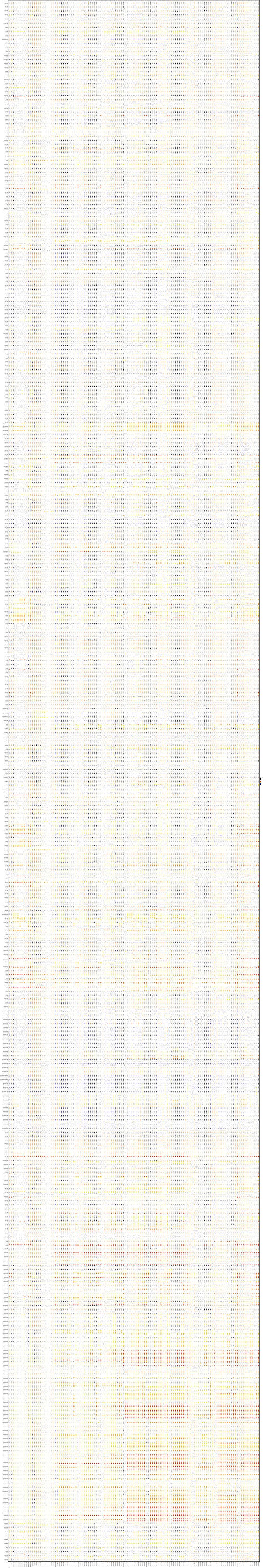

Supplement: Supplementary file 1 [file DataSheet_1.pdf]
